# Ultrasound characterization of the mastoid for detecting middle ear effusion: A preliminary clinical validation

**DOI:** 10.1038/srep27777

**Published:** 2016-06-09

**Authors:** Chin-Kuo Chen, Jui Fang, Yung-Liang Wan, Po-Hsiang Tsui

**Affiliations:** 1 Department of Otolaryngology—Head and Neck Surgery, Chang Gung Memorial Hospital and Chang Gung University, Taoyuan, Taiwan; 2 Ph.D. Program in Biomedical Engineering, College of Engineering, Chang Gung University, Taoyuan, Taiwan; 3 Department of Medical Imaging and Radiological Sciences, College of Medicine, Chang Gung University, Taoyuan, Taiwan; 4 Medical Imaging Research Center, Institute for Radiological Research, Chang Gung University and Chang Gung Memorial Hospital at Linkou, Taoyuan, Taiwan; 5 Department of Medical Imaging and Intervention, Chang Gung Memorial Hospital at Linkou, Taoyuan, Taiwan

## Abstract

Ultrasound detection of middle ear effusion (MEE) is an emerging technique in otolaryngology. This study proposed using ultrasound characterization of the mastoid to noninvasively measure MEE-induced mastoid effusion (ME) as a new strategy for determining the presence of MEE. In total, 53 patients were enrolled (Group I: normal, n = 20; Group II: proven MEE through both otoscopy and tympanometry, n = 15; Group III: patients with MEE having effusions observed during grommet surgery, n = 18). A 2.25-MHz delay-line transducer was used to measure backscattered signals from the mastoid. The Nakagami parameter was estimated using the acquired signals to model the echo amplitude distribution for quantifying changes in the acoustic structures of mastoid air cells. The median Nakagami parameter and interquartile range were 0.35 (0.34–0.37) for Group I, 0.39 (0.37–0.41) for Group II, and 0.43 (0.39–0.51) for Group III. The echo amplitude distribution observed for patients with MEE was closer to Rayleigh distribution than that without MEE. Receiver operating characteristic (ROC) curve analysis further revealed that the area under the ROC was 0.88, sensitivity was 72.73%, specificity was 95%, and accuracy was 81.13%. The proposed method has considerable potential for noninvasive and comfortable evaluation of MEE.

Otitis media, an inflammatory process of the middle ear, is one of the most common infections^1^,^2^. Acute otitis media may cause severe symptoms (e.g., fever, otalgia, and otorrhea) and is often accompanied by middle ear effusion (MME) because of a block of the eustachian tube caused by the swelling of lymphoid tissues. MEE may impair hearing and thereby affect speech development and the quality of life^3^,^4^. Clinically, otoscopy and tympanometry are commonly used for detecting MEE^5^; however, these methods require patients to remain motionless and quiet, and the diagnostic accuracy depends on operator experience^6^,^7^,^8^. Therefore, additional techniques that may assist in determining the presence of MEE are required.

Ultrasound, defined as sound waves with frequencies higher than 20 kHz, is widely used in various medical applications because of its cost efficiency, nonionizing radiation, simple signal processing, and real-time capability. Several studies have reported the advantages of ultrasound in assessing MEE^9^,^10^,^11^,^12^,^13^,^14^. The previously proposed ultrasound technique involves positioning an ultrasound probe in front of the tympanic membrane through the external ear canal. Signal waveforms of ultrasonic echoes from the eardrum differ depending on whether the middle ear is filled with fluid. In a normal air-filled eardrum, an echo is reflected only from the eardrum itself, whereas in a fluid-filled eardrum, a second echo emanates from the bony medial wall of the tympanic membrane. Although this ultrasound technology provides crucial clues associated with MEE, it is relatively invasive and requires injecting sterile water into the external ear canal to provide a coupling medium for ultrasound propagation. Conscious patients may be intolerant to this approach, thus limiting its clinical applicability^14^. Moreover, an increase in the thickness of the tympanic membrane because of postsurgery or inflammation may cause the attenuation of ultrasound, which is another possible reason affecting ultrasound measurements because of a poor signal-to-noise ratio.

To resolve the aforementioned limitations, ultrasound tissue characterization of the mastoid, which is located behind the ear, may provide a favorable opportunity to achieve noninvasive and routinely usable ultrasound techniques for clinical MEE detection. The rationale for proposing this idea is as follows. Mastoid cells are air pockets in a honeycomb-shaped bone structure and are connected with the middle-ear cavity; these cells are altered in most ears with MEE^15^,^16^,^17^. Some studies have specifically reported fluid accumulation in the mastoid cells of patients with MEE^18^,^19^, and this phenomenon can be visualized through computed tomography (CT), as shown in Fig. 1. Mastoid effusion (ME) can be a useful indicator of MEE. The mastoid is located under the skin; therefore, an ultrasound transducer can be placed directly on the mastoid to measure the echo signals for detecting ME. Moreover, MEE-induced effusions in the mastoid changes the acoustic impendence of air cells, thus changing the intensity of ultrasound signals reflected from the mastoid. This can be supported by our previous study in human cadavers, which showed that ME changes the amplitude of ultrasound signals^20^.

However, using the intensity analysis of ultrasound echo alone may be insufficient to characterize the mastoid because air cells of various shapes and sizes are randomly distributed in the mastoid. In a relatively complex mastoid structure, the interaction between air cells and the incident wave tends to produce ultrasound scattering; thus, the received ultrasound echoes backscattered from the air cells may be considered random signals. Different scattering structures result in different properties of ultrasound backscattered signals^21^. Based on the randomness of ultrasound backscattering, statistical distributions have been widely used to model the echo amplitude distribution for tissue characterization^21^. Among all possibilities, the Nakagami parameter of the Nakagami distribution is a relatively simple and general parameter to quantify the echo amplitude distribution^22^,^23^,^24^,^25^. In brief, the Nakagami parameter is estimated using the second and fourth statistical moments of signal amplitude data (i.e., envelope signal), which are typically obtained by considering the absolute value of the Hilbert transform of ultrasound backscattered signals^23^,^24^,^25^. Furthermore, as the Nakagami parameter varies from 0 to 1, the echo amplitude distribution changes from a pre-Rayleigh to a Rayleigh distribution; a Nakagami parameter of >1 represents that the echo amplitude distribution conforms to a post-Rayleigh distribution^22^. Recently, the Nakagami parameter has been widely used in soft-tissue characterization of, for example, cataracts^26^, blood^27^, liver^28^,^29^, breast^30^, and thrombus^31^. The Nakagami parameter is also used in modeling the echo amplitude distribution of bony tissues^32^,^33^. Therefore, we assumed that the Nakagami parameter can quantify the acoustic structure of the mastoid and correlated it with clinical MEE.

In this study, we explored the feasibility of using the Nakagami parameter to characterize the mastoid for detecting MEE. We first established a portable single-crystal ultrasound system equipped with a 2.25-MHz delay-line transducer. We enrolled 53 participants, including normal cases (Group I) and patients with MEE (Groups II and III). MEE in participants in Groups I and II were clinically examined through both otoscopy and tympanometry. Furthermore, MEE in patients in Group III was proven according to effusions observed during grommet surgery. For each participant, ultrasound backscattered signals were acquired from the mastoid for estimating the Nakagami parameter. The results revealed that the Nakagami parameter of the mastoid efficiently discriminated between patients without and with MEE. Finally, this study discussed clinical values of the proposed method for the future diagnosis of MEE.

## Results

**Figure 2:**
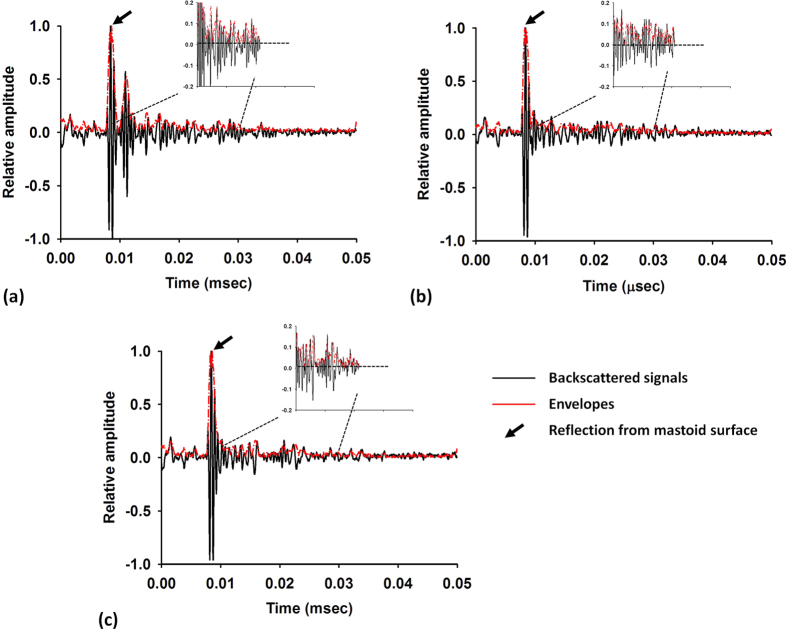
Typical ultrasound backscattered signals measured from the mastoid. The black line indicates the backscattered echoes, and the red line indicates envelopes (i.e., echo amplitude data). (**a**) Group I: normal cases; (**b**) Group II: clinically proven MEE through otoscopy and tympanometry; (**c**) Group III: MEE proven according to the findings of effusions during grommet surgery.

Figure 2 shows ultrasound echoes and the corresponding envelope signals (i.e., echo amplitude data) measured from the mastoid. The strong reflection signals from the mastoid surface are indicated by black arrows. The signals between 0 and 0.01 ms are background signals in the delay-line material of the transducer, and those after 0.01 ms are contributed by air cells in the mastoid. The amplitude of the backscattered signals tended to be large and exhibited a high fluctuation for participants in Group I. The backscattered echoes measured from the mastoids in patients in Groups II and III tended to be less intense and have a low degree of variance in their signal amplitude. The difference in the signal intensity and waveform between different groups may be attributed to ME, which can be treated as the change in the acoustic structures of the mastoid caused by MEE.

**Figure 3:**
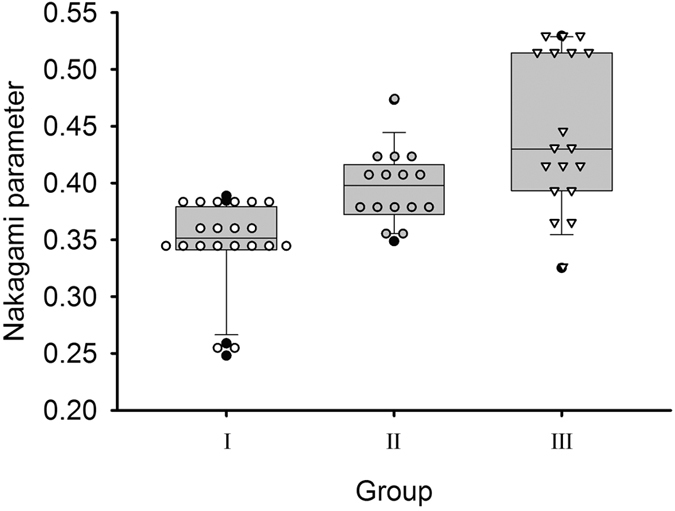
Nakagami parameter obtained from different groups. The Nakagami parameter increased with the increasing effusion stage from Group I to Group III (*r* = 0.66; *p* < 0.001). A significant difference was observed between Groups I and II (*p* < 0.05). No significant difference between Groups II and III (*p* = 0.08).

To confirm the observations, ultrasound envelope signals received between 0.01 and 0.03 ms were used to estimate the Nakagami parameter, as shown in Fig. 3. Data were expressed as median and interquartile range (IQR). The Nakagami parameter increased with the increasing effusion stage from Groups I to III (Pearson correlation coefficient, *r* = 0.66; probability value, *p* < 0.001). The median Nakagami parameter was 0.35 (IQR: 0.34–0.37) for Group I, 0.39 (IQR: 0.37–0.41) for Group II, and 0.43 (IQR: 0.39–0.51) for Group III. This result indicated that the echo amplitude distribution obtained for patients with MEE was closer to Rayleigh distribution than that obtained for participants without MEE. A significant difference was observed between Groups I/II and I/III (*p* < 0.05).

**Figure 4:**
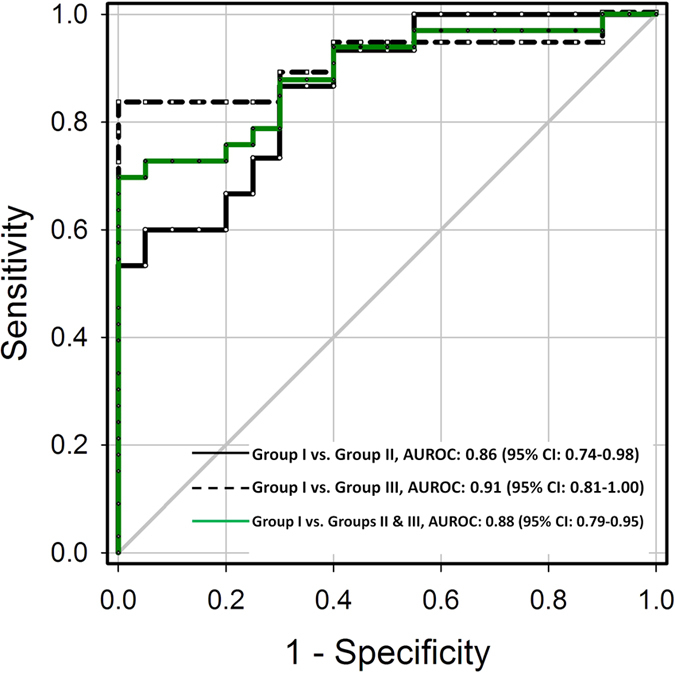
ROC analysis for diagnosing MEE. The AUROC at 95% CI was 0.86 (0.74–0.98) and 0.91 (0.81–1.00) for discriminating between Groups I/II and Groups I/III, respectively. Combining MEE data of Groups II and III, the AUROC at 95% CI was 0.88 (0.79–0.95), diagnostic sensitivity was 72.73%, specificity was 95%, and accuracy was 81.13%.

To preliminarily evaluate the diagnostic value of the proposed method in determining MEE, the receiver operating characteristic curves (ROCs) for diagnosing different groups are shown in Fig. 4. The area under ROC (AUROC) at 95% confidence interval (CI) was 0.86 (0.74–0.98) and 0.91 (0.81–1.00) for discriminating between Groups I/II and Groups I/III, respectively. No significant difference was observed between Groups II and III (*p* = 0.08); therefore, we combined the MEE data of Groups II and III for ROC analysis. The AUROC at 95% CI was 0.88 (0.79–0.95), diagnostic sensitivity was 72.73%, specificity was 95%, and accuracy was 81.13%. The performance profile for using the Nakagami parameter in MEE assessment is shown in Table 1.

## Discussion

Ultrasound characterization of the mastoid to determine the presence of MEE is a novel noninvasive approach. In this study, we validated this idea by using clinical data. The results obtained from the participants with and without MEE revealed that the behavior of ultrasound backscattering from the mastoid depends on MEE. Moreover, MEE-induced ME alters the echo amplitude distribution of the mastoid, thus increasing the corresponding Nakagami parameter. According to our review of relevant literature, this study is the first to validate the correlation between the Nakagami parameter (a quantitative estimate of ultrasound backscattered statistics) of the mastoid and MEE. Importantly, by using the mastoid as an acoustic window for ultrasound measurements, the proposed method provides a noninvasive technique for MEE evaluation.

The mastoid composed of air cells is one part of the temporal bone. The propagation of an ultrasound wave is more complex through bones than through soft tissues. Soft tissues behave like fluids with weak variations in their material properties^34^. However, bone tissues, which are solid and support both compressive and shear wave propagations, have anisotropic heterogeneous porous structures with strong variations in the properties of scatterers^35^. The echo amplitude distribution measured from tissues can be classified into three types^23^: (i) Rayleigh distribution caused by a large number of randomly distributed scatterers in the resolution cell of the transducer (e.g., a homogeneous medium); (ii) pre-Rayleigh distribution (with a phase lead compared with Rayleigh statistics) because of a low scatterer concentration or scatterers in the resolution cell having randomly varying scattering cross-sections with a comparatively high degree of variance (e.g., inhomogeneous or heterogeneous medium); (iii) post-Rayleigh distribution (with a phase lag compared with Rayleigh) caused by a resolution cell containing periodically located scatterers in addition to randomly distributed scatterers (e.g., scatterer clustering). A heterogeneous porous structure in a bone tissue is similar to condition (ii), which may explain why the Nakagami parameter of the mastoids are smaller than 1 (i.e., pre-Rayleigh distribution). In particular, the temporal bone is a hard tissue; thus, multiple scattering has to be considered^36^,^37^. Multiple scattering is also a possible factor causing a high degree of variance in the echo signal amplitude, thus making the backscattered signals follow pre-Rayleigh distribution.

At present, no relevant study or theories have discussed why effusion affects the echo amplitude distribution of the mastoid. We believe that the effusion changes the acoustic impedance, which affects the acoustic interactions between ultrasound waves and the mastoid structures. Effusion in the mastoid reduces the magnitude of the discontinuities in the acoustic impedance between each air cell. Consequently, the degree of the signal fluctuation decreases, which results in a shift of the echo amplitude distribution toward Rayleigh distribution and an increase in the Nakagami parameter, as supported by the present clinical findings (Fig. 3). The Nakagami parameters of Group III were slightly higher than those of Group II because patients who clinically require grommet surgery typically exhibit severe effusion in the middle ear cavity^38^,^39^. Thus, more fluids may accumulate in their mastoid cells to yield larger estimates of the Nakagami parameter.

Compared with the results of previous studies^10^,^11^,^13^,^14^ the proposed Nakagami approach uses the mastoid as an acoustic window, thus allowing physicians to simply position an ultrasound probe on the mastoid and transmit an ultrasound pulse into the mastoid to determine MEE. Such a noninvasive technique allows patient comfort during examination. In particular, the Nakagami parameter involves low computational complexity; thus, its algorithm can be easily integrated into any hardware to facilitate real-time computation^40^. In the future, the proposed method and algorithmic scheme can be used to develop a small, office-based ultrasound measurement device as a rapid screening tool for detecting MEE.

The study has some limitations. First, the performance of the Nakagami parameter on characterizing different types of mastoids (e.g., pneumatic, sclerotic, or diploetic type) has not been explored. The proposed method may not efficiently determine MEE when the mastoid structures are not completely developed. Second, the Nakagami parameter estimation depends on the frequency^41^. beam focusing^24^. and estimator^29^. These effects must be further investigated for optimizing the hardware and software parameters in a clinical setting. Third, mild MEE may not induce ME; therefore, the performance of the proposed technique in the early assessment of MEE may be limited. In future work, using CT or magnetic resonance imaging (MRI) to simultaneously confirm the statuses of ME and MEE may be a useful strategy if the stage of MEE (e.g., mild, moderate, and severe) needs to be further identified.

## Conclusion

This study proposed ultrasound characterization of the mastoid as a noninvasive approach for determining the presence of MEE. The acoustic structure of the mastoid is quantified by the Nakagami parameter to reflect effusion-induced changes in acoustic impedance. The clinical results revealed a higher Nakagami parameter for mastoids of patients with MEE than for participants without MEE; this result represents that echo amplitude distributions obtained for patients with MEE tend to be closer to Rayleigh distribution than those obtained for participants without MEE. The ROC analysis revealed that the diagnostic accuracy of the Nakagami parameter in assessing MEE was 81.13%. Furthermore, compared with the conventional ultrasound technique, in which a probe is inserted into the external ear canal, the proposed method based on the Nakagami approach and a single-crystal ultrasound system ensures patient comfort, this increasing its clinical applicability; hence, it may become a routinely usable noninvasive tool for a comfortable evaluation of MEE.

## Materials and Methods

### Patient recruitment

Clinical measurements were performed at Chang Gung Memorial Hospital at Linkou, Taiwan, whose institutional review board approved this study. All the experimental methods were carried out in accordance with the approved guidelines. Informed consent was obtained from all patients and their relatives. In total, 53 patients were enrolled in the Department of Otolaryngology–Head and Neck Surgery between April 2013 and April 2014. According to their clinical diagnoses, the participants were categorized into three groups: normal cases (n = 20; Group I) and patients with MEE (n = 15; Group II; n = 18, Group III). MEE for the participants in Groups I and II was clinically examined through both otoscopy and tympanometry by an experienced otolaryngologist. The patients in Group III were scheduled to undergo grommet surgery, which involves effusion draining through a ventilation tube inserted into the eardrum. MEE in the patients of Group III was proven on the basis of observations of effusions during grommet surgery.

### Ultrasound system

**Figure 5:**
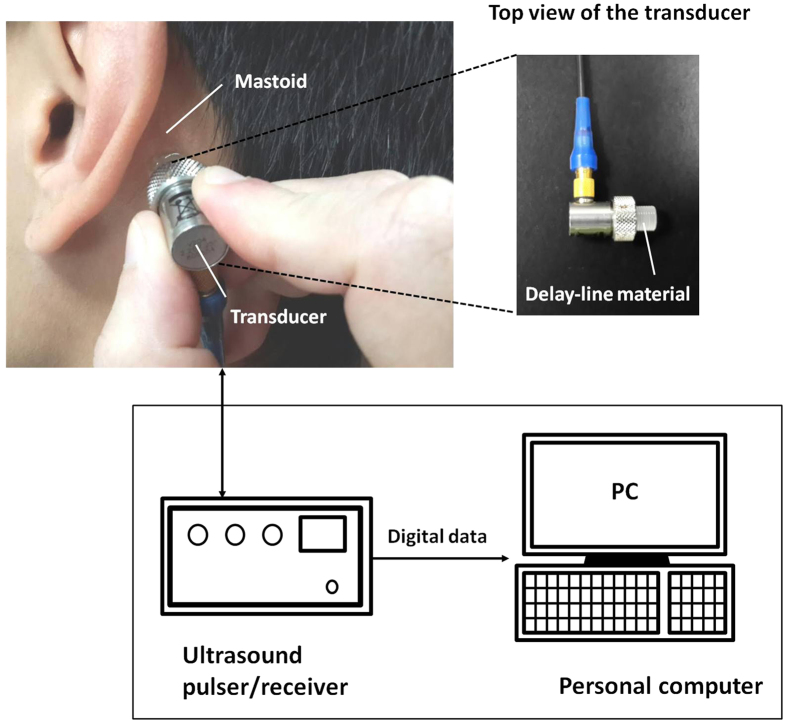
Illustration and the actual image of the single-crystal ultrasound system used in the study. The system comprises a 2.25-MHz delay-line transducer, portable ultrasound pulser–receiver, and personal computer.

**Figure 6:**
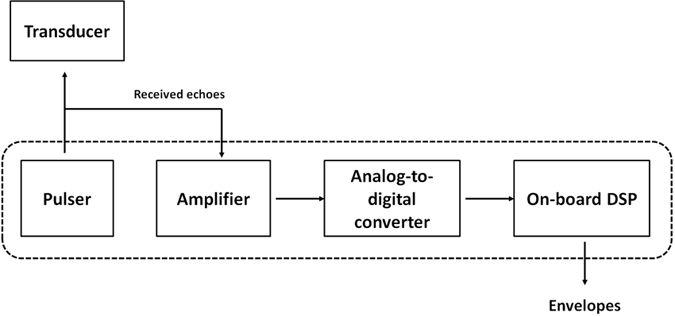
Schematics of the pulser-receiver, which comprises a pulser, an amplifier, an analog-to-digital converter, and an on-board digital signal processing chip.

Figure 5 shows the ultrasound system used to measure the backscattered signals from the mastoid air cells. The system included a portable ultrasound pulser–receiver (Model USB-UT350, US Ultratek, Inc., Martinez, CA, USA) with a USB interface connected to a computer operating on the environment of Windows XP. The pulser–receiver consists of a pulser, an amplifier (gain: 0 to 80 dB; receiving bandwidth: 0.6 to 18 MHz), a 8-bit analog-to-digital converter (ADC) with a maximum sampling rate of 50 MHz, and an on-board digital signal processing (DSP) chip, as shown in Fig. 6. The pulser generates an electrical pulse to drive an ultrasonic transducer for transmitting ultrasound waves. The same transducer receives echoes that are then amplified by the amplifier, digitalized by the ADC, and demodulated by the DSP chip for real-time envelope detection. Using the software development kit provided by the manufacturer, a user interface was made using the LabVIEW software (Version 12, National Instruments Corporation, Austin, TX, USA) to allow real-time signal observation and data acquisition operation on the computer.

A delay-line single-element transducer (V204-RM, Panametrics-NDT, Waltham, MA, USA) with a central frequency of 2.25 MHz and bandwidth of 60% was used. The delay-line transducer is a key component when transmitting ultrasound into the mastoid for receiving the backscattered signals. The transducer was driven by a high-voltage excitation signal generated from a pulser and thus the excitation signal and backscattered echo from the mastoid may overlap during data acquisition, complicating the observation of the behaviors of the backscattered signals from the mastoid air cells. Under this condition, a delay-line transducer is highly suitable for mastoid measurements^42^. Delay-line transducers are of a single-element type, specifically designed to incorporate a short piece of plastic or epoxy material in front of the transducer surface. Using delay-line materials, the excitation pulse and echoes reflected from the mastoid can be effectively separated.

### Ultrasound measurements

**Figure 7:**
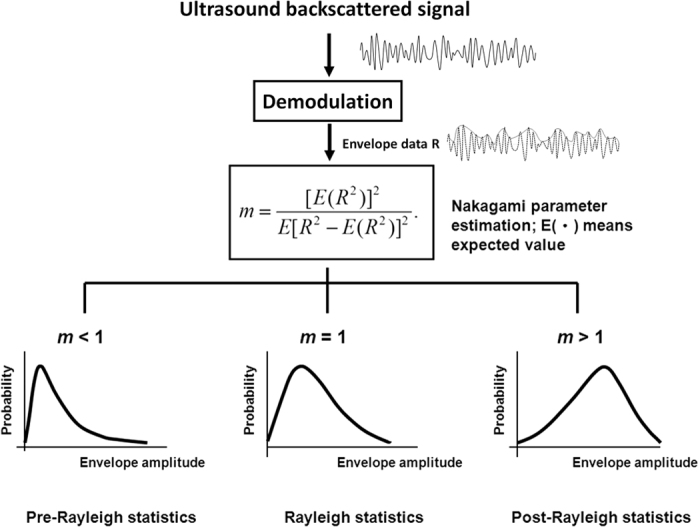
Algorithmic scheme used in this study. Ultrasound backscattered signals measured from the mastoid were demodulated using Hilbert transform to obtain the corresponding envelopes for estimating the Nakagami parameter. The Nakagami parameter was estimated from the second and fourth moments of envelope signals (i.e., echo amplitude data).

Before each patient underwent MEE examination, ultrasound measurements were performed by an experienced radiologist. The radiologist was blinded to the examination report of each patient. As shown in Fig. 5, the transducer was positioned on the mastoid, and ultrasound coupling gel was applied between the mastoid and transducer to improve wave propagation. Five independent measurements were performed to acquire the backscattered signals (gain: 60 dB; sampling rate: 25 MHz; length of each acquired signal: 0.05 ms) Refer to Fig. 7. The acquired backscattered envelopes were used to estimate the Nakagami parameter *m* associated with the Nakagami distribution as follows^22^:


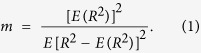


Where *R* is the envelope of backscattered signals and E (·) denotes the statistical mean. For each patient, the estimated Nakagami value was computed by averaging the results obtained from each measurement.

### Statistical analysis

The Nakagami parameters in the three groups were compared to determine the correlation between the Nakagami parameter and MEE. Data were expressed as median and IQR. The Pearson correlation coefficient *r* and probability value *p* were calculated to evaluate the correlation between the Nakagami parameter and MEE. Moreover, independent *t* tests were performed to compare the difference of the Nakagami parameter between each group; *p* < 0.05 was considered statistically significant. To preliminarily evaluate the diagnostic value of the Nakagami parameter in determining the presence of MEE, ROC analysis at 95% CI was performed to obtain the AUROC. Sensitivity, specificity, and accuracy were also reported. All statistical analyses were conducted using SigmaPlot software (Version 12.0, Systat Software, Inc., CA, USA).

## Additional Information

**How to cite this article**: Chen, C.-K. *et al.* Ultrasound characterization of the mastoid for detecting middle ear effusion: A preliminary clinical validation. *Sci. Rep.* **6**, 27777; doi: 10.1038/srep27777 (2016).

## Figures and Tables

**Figure 1:**
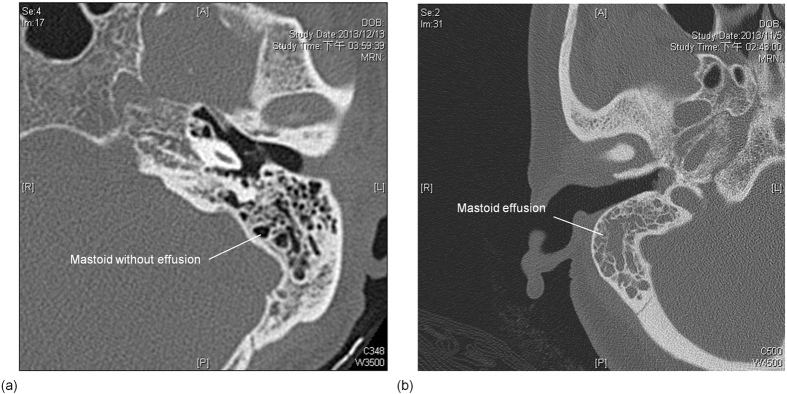
Typical computed tomography images of mastoids for patients without (left) and with MEE (right) captured at Chang Gung Memorial Hospital at Linkou, Taiwan. Air cells in the mastoid correspond to gray shading, representing a water-based medium (effusion).

**Table 1:** Clinical performance of the ultrasound Nakagami parameter in assessing MEE.

Parameter	Group I vs. Group II	Group I vs. Group III	Group I vs. Groups II & III
Cutoff value	0.3655	0.3888	0.3846
Sensitivity, %	86.67 (59.54 to 98.34)	83.33 (58.58 to 96.42)	72.73 (54.48 to 86.70)
Specificity, %	65.00 (40.78 to 84.61)	95.00 (75.13 to 99.87)	95.00 (75.13 to 99.87)
Accuracy, %	77.14	92.10	81.13
LR+	2.4763	16.6660	14.5460
LR−	0.2051	0.1755	0.2871
AUROC	0.86 (0.74–0.98)	0.91 (0.81–1.00)	0.88 (0.79–0.95)

LR+: positive likelihood ratio, LR−: negative likelihood ratio, AUROC: area under the receiver operating characteristics curve.
